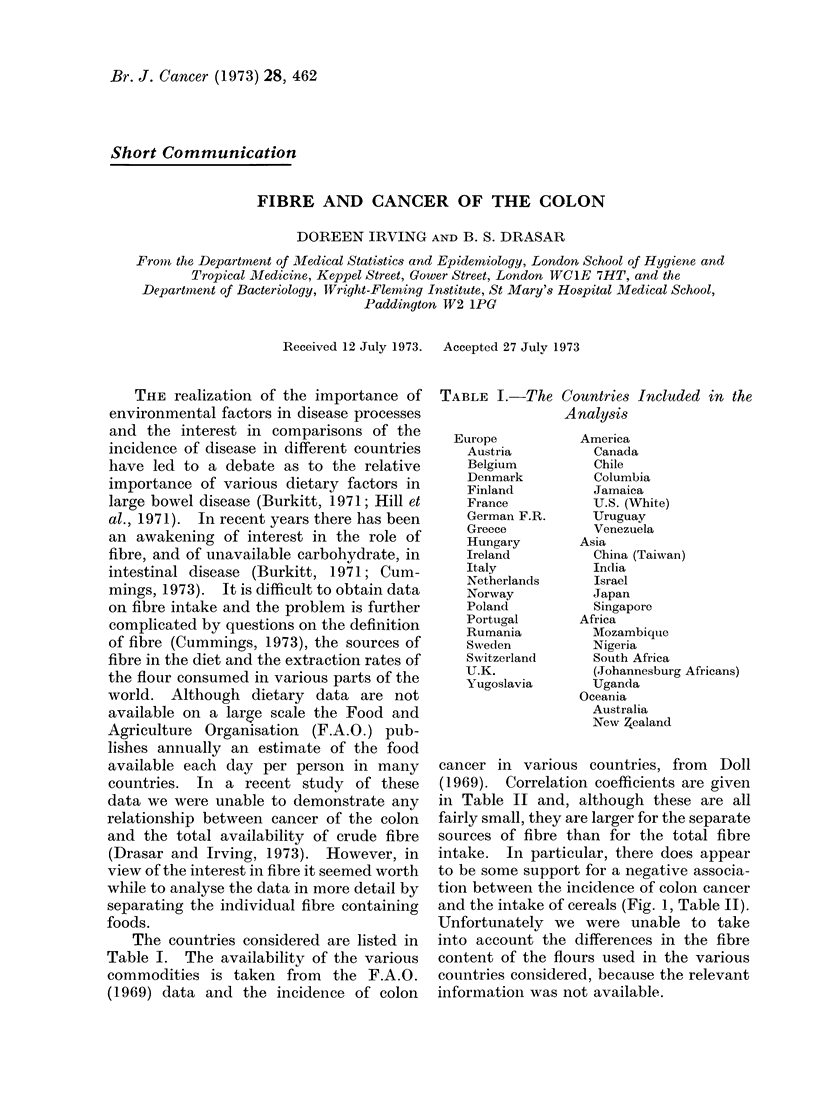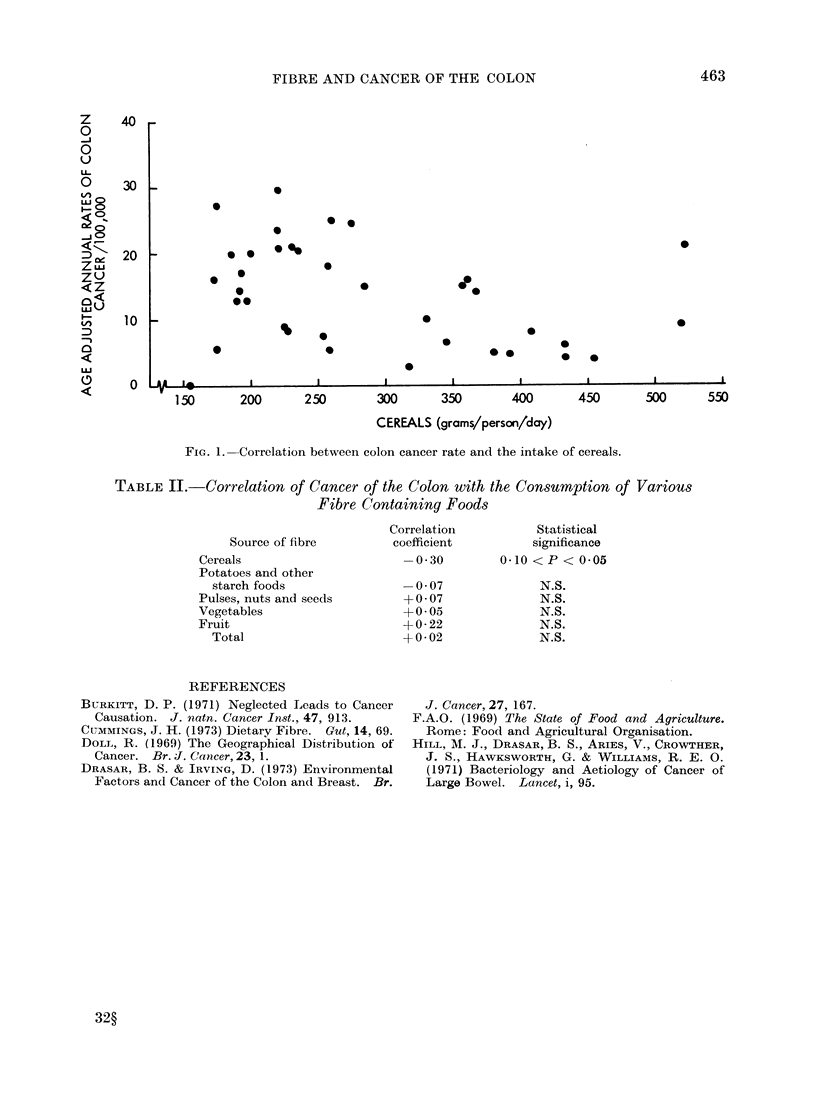# Fibre and cancer of the colon.

**DOI:** 10.1038/bjc.1973.173

**Published:** 1973-11

**Authors:** D. Irving, B. S. Drasar


*Br. J. Cancer* (1973) **28**, 462

***Short Communication***

### FIBRE AND CANCER OF THE COLON

DOREEN IRVING AND B. S. DRASAR

*From the Department of Medical Statistics and Epidemiology, London School of Hygiene and Tropical Medicine, Keppel Street, Gower Street, London WC1E 7HT, and the Department of Bacteriology, Wright-Fleming Institute, St Mary's Hospital Medical School, Paddington W2 1PG*

Received 12 July 1973. Accepted 27 July 1973

THE realization of the importance of environmental factors in disease processes and the interest in comparisons of the incidence of disease in different countries have led to a debate as to the relative importance of various dietary factors in large bowel disease (Burkitt, 1971; Hill *et al*., 1971). In recent years there has been an awakening of interest in the role of fibre, and of unavailable carbohydrate, in intestinal disease (Burkitt, 1971; Cummings, 1973). It is difficult to obtain data on fibre intake and the problem is further complicated by questions on the definition of fibre (Cummings, 1973), the sources of fibre in the diet and the extraction rates of the flour consumed in various parts of the world. Although dietary data are not available on a large scale the Food and Agriculture Organisation (F.A.O.) publishes annually an estimate of the food available each day per person in many countries. In a recent study of these data we were unable to demonstrate any relationship between cancer of the colon and the total availability of crude fibre (Drasar and Irving, 1973). However, in view of the interest in fibre it seemed worth while to analyse the data in more detail by separating the individual fibre containing foods.

The countries considered are listed in Table I. The availability of the various commodities is taken from the F.A.O. (1969) data and the incidence of colon cancer in various countries, from Doll (1969). Correlation coefficients are given in Table II and, although these are all fairly small, they are larger for the separate sources of fibre than for the total fibre intake. In particular, there does appear to be some support for a negative association between the incidence of colon cancer and the intake of cereals (Fig. 1, Table II). Unfortunately we were unable to take into account the differences in the fibre content of the flours used in the various countries considered, because the relevant information was not available.

TABLE I.—*The Countries Included in the Analysis*

| | |
|---|---|
| Europe | America |
| Austria | Canada |
| Belgium | Chile |
| Denmark | Columbia |
| Finland | Jamaica |
| France | U.S. (White) |
| German F.R. | Uruguay |
| Greece | Venezuela |
| Hungary | Asia |
| Ireland | China (Taiwan) |
| Italy | India |
| Netherlands | Israel |
| Norway | Japan |
| Poland | Singapore |
| Portugal | Africa |
| Rumania | Mozambique |
| Sweden | Nigeria |
| Switzerland | South Africa |
| U.K. | (Johannesburg Africans) |
| Yugoslavia | Uganda |
| | Oceania |
| | Australia |
| | New Zealand |

FIG. 1.—Correlation between colon cancer rate and the intake of cereals.

TABLE II.—*Correlation of Cancer of the Colon with the Consumption of Various Fibre Containing Foods*

| Source of fibre | Correlation coefficient | Statistical significance |
|---|---|---|
| Cereals | −0·30 | $0{\cdot}10 < P < 0{\cdot}05$ |
| Potatoes and other starch foods | −0·07 | N.S. |
| Pulses, nuts and seeds | +0·07 | N.S. |
| Vegetables | +0·05 | N.S. |
| Fruit | +0·22 | N.S. |
| Total | +0·02 | N.S. |

### REFERENCES

BURKITT, D. P. (1971) Neglected Leads to Cancer Causation. *J. natn. Cancer Inst.*, **47**, 913.

CUMMINGS, J. H. (1973) Dietary Fibre. *Gut*, **14**, 69.

DOLL, R. (1969) The Geographical Distribution of Cancer. *Br. J. Cancer*, **23**, 1.

DRASAR, B. S. & IRVING, D. (1973) Environmental Factors and Cancer of the Colon and Breast. *Br. J. Cancer*, **27**, 167.

F.A.O. (1969) *The State of Food and Agriculture.* Rome: Food and Agricultural Organisation.

HILL, M. J., DRASAR, B. S., ARIES, V., CROWTHER, J. S., HAWKSWORTH, G. & WILLIAMS, R. E. O. (1971) Bacteriology and Aetiology of Cancer of Large Bowel. *Lancet*, i, 95.